# Extracellular cold inducible RNA-binding protein mediates binge alcohol-induced brain hypoactivity and impaired cognition in mice

**DOI:** 10.1186/s10020-019-0092-3

**Published:** 2019-05-30

**Authors:** Asha Jacob, Yilong Ma, Elham Nasiri, Mahendar Ochani, Joseph Carrion, Shichun Peng, Max Brenner, Patricio T. Huerta, Ping Wang

**Affiliations:** 1 Immunology and Inflammation, Institute of Molecular Medicine, The Feinstein Institutes for Medical Research, Northwell Health, Manhasset, NY USA; 2 Center for Neurosciences, Institute of Molecular Medicine, The Feinstein Institutes for Medical Research, Northwell Health, Manhasset, NY USA; 3 Laboratory of Immune & Neural Networks, Institute of Molecular Medicine, The Feinstein Institutes for Medical Research, Northwell Health, Manhasset, NY USA; 4 Department of Molecular Medicine, Donald and Barbara Zucker School of Medicine at Hofstra/Northwell, Hempstead, NY USA; 5 Departments of Surgery and Molecular Medicine, Donald and Barbara Zucker School of Medicine at Hofstra/Northwell, Hempstead, NY USA

**Keywords:** CIRP, microPET, Fluorodeoxyglucose, Spatial memory, Hippocampus, Amygdala

## Abstract

**Background:**

Alcohol abuse affects the brain regions responsible for memory, coordination and emotional processing. Binge alcohol drinking has shown reductions in brain activity, but the molecular targets have not been completely elucidated. We hypothesized that brain cells respond to excessive alcohol by releasing a novel inflammatory mediator, called cold inducible RNA-binding protein (CIRP), which is critical for the decreased brain metabolic activity and impaired cognition.

**Methods:**

Male wild type (WT) mice and mice deficient in CIRP (CIRP^−/−^) were studied before and after exposure to binge alcohol level by assessment of relative brain glucose metabolism with fluorodeoxyglucose (^18^FDG) and positron emission tomography (PET). Mice were also examined for object-place memory (OPM) and open field (OF) tasks.

**Results:**

Statistical Parametric Analysis (SPM) of ^18^FDG-PET uptake revealed marked decreases in relative glucose metabolism in distinct brain regions of WT mice after binge alcohol. Regional analysis (post hoc) revealed that while activity in the temporal (secondary visual) and limbic (entorhinal/perirhinal) cortices was decreased in WT mice, relative glucose metabolic activity was less suppressed in the CIRP^−/−^ mice. Group and condition interaction analysis revealed differing responses in relative glucose metabolism (decrease in WT mice but increase in CIRP^−/−^ mice) after alcohol in brain regions including the hippocampus and the cortical amygdala where the percent changes in metabolic activity correlated with changes in object discrimination performance. Behaviorally, alcohol-treated WT mice were impaired in exploring a repositioned object in the OPM task, and were more anxious in the OF task, whereas CIRP^−/−^ mice were not impaired in these tasks.

**Conclusion:**

CIRP released from brain cells could be responsible for regional brain metabolic hypoactivity leading to cognitive impairment under binge alcohol conditions.

## Introduction

Binge drinking is a popular mode of alcohol intake in young adults and the worldwide prevalence of such occurrence is about 16% (Global status report on alcohol and health 2018, Geneva: World Health Organization, https://www.who.int/substance_abuse/publications/global_alcohol_report/en/). The National Institute of Alcohol Abuse and Alcoholism (NIAAA) defines binge drinking as ≥4 drinks for a woman and ≥ 5 drinks for a man in about 2 h. A blood alcohol concentration (BAC) of 17.4 mM (0.08%) indicates excessive drinking and it is the legal BAC limit for driving in the US. Alcohol abuse negatively affects nearly all body tissues, and particularly the brain regions essential in memory, coordination and emotional processing (Guerri and Pascual 2010; Jacobus and Tapert 2013). Assessment of glucose metabolism has long been used to measure brain synaptic activity and function. Measurement of relative glucose utilization with fluorodeoxyglucose (^18^FDG) and positron emission tomography (PET) has the potential to detect early brain dysfunction prior to any abnormal findings in neuropsychological testing (Thanos et al. 2008). ^18^FDG-PET imaging has been utilized to acquire neural snapshots of glucose metabolism in alcoholics and non-alcoholic healthy subjects (Volkow et al. 1992; Volkow et al. 1990). Oral administration of moderate to high doses of alcohol to non-alcoholic healthy adults markedly reduces their relative brain glucose metabolism (Schreckenberger et al. 2004; Volkow et al. 2006). However, the molecular targets for high alcohol in the brain have not been completely elucidated.

Cold inducible RNA-binding protein (CIRP) is a 172 amino acid protein belonging to a family of cold shock proteins (Nishiyama et al. 1997). It consists of a conserved N-terminal RNA binding domain, and a C-terminal glycine rich domain. Recent work has identified a novel function of CIRP in that, upon cellular stress, it is released from the cell and functions as a danger associated molecular pattern (DAMP) that promotes inflammation. Thus, extracellular CIRP represents a novel inflammatory mediator in stress conditions (Qiang et al. 2013). Functional brain imaging studies reveal that binge alcohol drinking in human adolescents causes structural alterations in distinct brain regions including the prefrontal cortex and the hippocampus (De Bellis et al. 2000; De Bellis et al. 2005). These structural anomalies have been correlated with cognitive impairment and neurophysiological abnormalities in young binge drinkers (De Bellis et al. 2000; De Bellis et al. 2005). Therefore, we hypothesize that binge alcohol increases the release of CIRP from brain cells and leads to decreased metabolic activity in regions associated with memory formation and cause impaired cognition. In the present study, to determine whether CIRP influences alcohol-mediated changes in brain metabolic activity, we exposed WT mice and mice deficient in CIRP (CIRP^−/−^) to binge levels of alcohol followed by ^18^FDG-PET imaging to assess relative brain glucose metabolism. The same cohort of mice was assessed behaviorally with the object place memory (OPM) and open field (OF) tasks. In an additional cohort of WT and CIRP^−/−^ mice, a test-retest study was conducted where the mice underwent an identical experimental protocol with the exception of being treated with normal saline instead of alcohol.

## Materials and methods

### Experimental animals

Breeder pairs of CIRP^−/−^ mice were a gift from Dr. Jun Fujita (Kyoto University, Japan). CIRP^−/−^ mice were 10x backcrossed on C57BL/6 background, and were bred in our animal facility (Qiang et al. 2013). Only male CIRP^−/−^ animals aged 10–12 weeks were used in this study. Male C57BL/6 mice aged 10–12 weeks (20–25 g, Taconic Labs, Albany, NY) were used as wild type (WT) controls. WT mice were acclimated to the environment for 5 days prior to any experiments. All mice were given food and water ad libitum. All animal protocols were performed based on the NIH Guide and Care of Laboratory Animals and were approved by the Institutional Animal Care and Use Committee of the Feinstein Institute for Medical Research.

### Model of binge alcohol in mice

WT and CIRP^−/−^ mice were anesthetized with 3% isoflurane inhalation and the right jugular vein was cannulated with a PE-10 catheter while maintaining anesthesia at 1–2%. Mice were infused with a bolus of 1.5 g per kg ethanol (23% v/v) in normal saline through the cannula and the other end of the catheter was connected via a mouse harness (SAI infusion technologies, Libertyville, IL) to an infusion pump (KD Scientific, Holliston, MA). The harness allowed free movement of the animal. Mice were allowed to recover from anesthesia and the initial bolus infusion for 1 h, after which they received 300 mg per kg per h ethanol via the infusion pump for 15 h totaling 6 g per kg alcohol. Afterwards, they were lightly re-anesthetized, the cannula was removed and the vein ligated.

### MicroPET imaging

Metabolic PET studies were performed following an established imaging protocol. Briefly, awake mice were given ^18^FDG (0.34–1.0 mCi/kg) intraperitoneally and were allowed unrestrained tracer uptake for 45 min. Mice were then anesthetized with 2–3% isoflurane inhalation and maintained at 1–2% while positioned in the Siemens Inveon PET scanner (Siemens AG, Munich, Germany). A 10-min static emission scan then was obtained within a field of view (FOV) of 12.7 cm, with a full width half maximum (FWHM) resolution of 1.2 mm at the center of FOV and a slice thickness of 0.796 mm. All mouse images were reconstructed using 3-dimensional Ordered Subset Expectation Maximization with Maximum A Priori (OSEM/3-D MAP). The resulting whole-body image had a matrix size of 128 mm X 128 mm X 159 mm and a pixel size of 0.776 X 0.776 X 0.796 cubic millimeters, which is consistent with the image resolution of the microPET scanner. Whole blood glucose values were taken after each scan using a commercially available kit. ^18^FDG-PET images underwent a series of preprocessing procedures described below.

### Acquisition and preprocessing of images

All mouse scan acquisitions were reconstructed using identical parameters and subsequently preprocessed on an Apple Mac Pro (Mac OS X 10.8.5, 2 × 2.93 Ghz 6-core intel Xeon microprocessors) using PMOD software version 3.3 (PMOD Technologies Ltd., Zurich, Switzerland). To extract the brain from the whole-body, each raw rodent image was cropped and re-oriented using a bounding box with a matrix size of 14.9 mm × 20.7 mm × 11.9 mm, and of the identical voxel size as the unprocessed image. For anatomical alignment, all reconstructed images were placed into the mouse brain stereotaxic space (Paxinos and Franklin 2008) using ^18^FDG-baseline template available on the PMOD website as a fusion tool (http://www.pmod.com/files/download/v36/doc/pbas/4996.htm). The ^18^FDG baseline template (Mirrione et al. 2007) was created using male C57/BL6 mice aged 2–5 months (24–34 g), and is based on a segmented MRI mouse brain dataset (Ma et al. 2005). The ^18^FDG template was opened in the PMOD reference window with activity set to milliCurie (mCi). Each reconstructed mouse scan file contained brain information for two mice, which necessitated loading image files in top-to-bottom configuration, and with the Z-axis perpendicular to the coronal slice. With these settings in place, manual co-registration of images proceeded in the following order: for each file, the first (top) mouse brain image was manually aligned to the reference ^18^FDG-template and saved. The second (bottom) mouse brain was then manually flipped into position by mirroring180° along the x-axis and registered to the ^18^FDG template. Implementing this method for all mouse images permitted individual registration in sagittal, coronal, and transverse anatomical planes. In addition, mouse brain images were “skull stripped” to remove all non-brain metabolic regions in the following manner: Each file was loaded together with an ^18^FDG-brain mask file (created using the ^18^FDG-baseline template) in the PMOD manual co-registration window to eliminate all pixel values outside of the brain image/^18^FDG-brain mask. The resulting images were converted into maps of standard uptake value (SUV) using injected dose and body weight for each animal to account for individual variability in injected radiotracer activity and weight. These images were then subsampled by approximately a factor of 10 to satisfy the theoretical requirement of Gaussian random field for subsequent brain mapping analysis described below with SPM.

### ^18^FDG-PET image analysis

PET images of SUV were analyzed using a mouse version of statistical parametric mapping software (SPM 5; Wellcome Department of Imaging Neuroscience, Institute of Neurology, London, UK) running in MATLAB 7.3 (The Mathworks Inc., MA). The baseline microPET brain images and those acquired post-alcohol were spatially aligned to each other, and then transformed into the standardized anatomical space using the ^18^FDG mouse brain template described above. Each image was normalized by its global value to minimize inter-individual variability and to increase statistical power. Analyses were performed on a voxel-by-voxel basis over the whole brain using general linear models implemented in SPM. A paired t-test model was first used to produce a set of brain regions in the WT mice showing highly significant decreases in globally normalized metabolic activity. Additionally, a two-way ANOVA was performed to reveal the effect of group x condition interaction. This analysis was conducted to examine explicitly the statistically significant group differences in regional metabolic activities between the two groups after treatment. The regions detected by each SPM run were then overlaid onto a mouse MRI brain template and identified using a mouse brain stereotaxic coordinates atlas (Paxinos and Franklin 2008). Globally normalized metabolic values were measured *post-hoc* in all PET images of WT and CIRP^−/−^ mice over each of the regions defined by separate SPM analysis above. Changes in regional metabolism between pre- and post-alcohol conditions were computed using percent change [(post alcohol – pre alcohol) × 100/pre-alcohol] in each animal.

### Behavioral testing

One week prior to behavioral assessment, mice were maintained on a reverse schedule of 12-h darkness (07:00 to 19:00) and 12-h light, with food and water ad libitum. For 3 days before testing, the mice were handled in daily sessions of 5–10 min. Animal handling and the initial testing were done during their dark circadian period. The mice were subjected to object-place memory (OPM) and open field (OF) tasks.

OPM testing was conducted as previously described (Faust et al. 2013). The apparatus consisted of a square base chamber (40 cm on each side X 60 cm high) built of polyvinyl chloride, which was painted gray. An orange-red light bulb (50 W) illuminated the chamber from above. An infrared sensitive camera (Marshall Electronics, model CV502-MB) was mounted above the chamber and connected to the video input for the behavior tracking software (Ethovision XT11.5, Noldus), which tracked the mouse position at 30 frames per sec. The chamber and the objects were cleaned with 70% ethanol between subjects and groups. The OPM task consisted of a familiarization trial (T1), a sample trial (T2) and a choice trial (T3) interspersed by 10-min intervals in the familiar square base chamber. For T1, mice were placed in the empty chamber for 10 min. For T2, mice explored the chamber for 10 min in the presence of two identical objects. For T3, mice explored the chamber for 10 min when one object remained at the same place as T2 (S) but the second object was moved to a different location in the chamber (M). The object exploration was measured with a software (Ethovision XT11.5, Noldus) algorithm that assigned a circular zone around each object and recorded the events in which the animal’s snout was in close proximity (< 1 cm) to the object. The sum of number of visits and the times spent on exploring both objects in T2 and T3 were also assessed. During T3, the discrimination ratio was calculated as “the time exploring the moved object (M) minus that of the stable object (S) over the times of the sum of both objects” or “[(M –S) / (M + S)]”.

For the OF task, the movements of the mice were automatically recorded for 10 min in the same chamber as the OPM task, and expressed as percent of total time the mice remained at the center of the chamber as opposed to the periphery, using an automated method (“arena setting tab” within Ethovision). The amount of time (sec) that the mice spent self-grooming was also measured during the OF task, using an automated method (“mouse behavior recognition module” within Ethovision).

### Experimental design

On Day 1 of the experiment, mice underwent pre-alcohol behavior tests in the behavior suite located adjacent to the microPET imaging suite. The cyclotron radiotracer preparation facility is also located adjacent to the microPET suite, and prior arrangements were made to have the radiotracer prepared for use at 9:00 AM. Ten minutes after the baseline behavior test, the unanesthetized and unrestrained mice were injected intraperitoneally with the ^18^FDG radiotracer in the microPET suite and 45 min allowed for ^18^FDG uptake to take place. Intraperitoneal (I.P.) injection of ^18^FDG is a valid alternative to intravenous (I.V.) injection, and it has been shown to have similar pharmacokinetics to IV administration in small animal studies (Wong et al. 2011). After allowing sufficient time for ^18^FDG uptake, mice were anesthetized with 3% isoflurane inhalation and placed on the microPET scanner with 1–2% isoflurane anesthesia maintained during scanning. After scanning, the mice were allowed to remain in the climate and light-controlled room within the microPET suite for 24–30 h to allow for radiotracer decay. On Day 2, mice were returned to the vivarium and anesthetized with 3% isoflurane inhalation, and the right jugular vein cannulated with a PE10 catheter while anesthesia was maintained at 1–2%. They were then infused with alcohol bolus (1.5 g per kg) and allowed 1 h for recovery from anesthesia and the bolus injection. Afterwards, the other end of the PE10 catheter was connected to an infusion pump and infused with alcohol (300 mg per kg per h) for 15 h. Mice were fitted with a harness allowing free movement of the animal during infusion (https://www.sai-infusion.com). At the end of 15 h, they were lightly anesthetized and the catheter was removed and the vein ligated. Immediately after the infusion, on Day 3, the mice were transferred to the microPET suite and injected intraperitoneally (IP) with ^18^FDG. During the ^18^FDG uptake, mice were subjected to post-alcohol behavior tests in an identical manner as the pre-alcohol behavior tests. After 45 min, the second microPET scan (post-alcohol) was acquired. Mice were then euthanized by CO_2_ asphyxiation (see Table 1 for a detailed timeline of each experiment). Using this timeline, experiments were performed in batches of 4 mice (2 WT and 2 CIRP^−/−^ mice) until completion of the study.

**Table 1:** Timeline for each experiment. Abbreviations: FDG, fluorodeoxyglucose; PET, positron emission tomography

DAY	Time	Procedure
1	9:00–10:00	Object Place Memory task #1
1	10:10–10:55	FDG injection and uptake
1	10:55–11:05	Pre-alcohol microPET scan under anesthesia
1–2	11:05–16:00	Tracer decay for 29–30 h
2	16:00–18:00	Vein cannulation, alcohol bolus (i.v.) under anesthesia
2	18:00–19:00	Recovery
2–3	19:00–9:00	Alcohol infusion via pump, without anesthesia
3	9:00–9:10	Vein ligation after 15 h under light anesthesia
3	9:20–10:05	FDG injection
3	9:20–10:05	Object Place Memory task #2 (assessed during FDG uptake)
3	10:05–10:15	Post-alcohol microPET scan under anesthesia
3	10:20	Euthanasia

A total of 14 mice per group were included in the study. Despite our attempt to utilize the same mice for behavioral assessment and microPET analysis, 3 WT mice and 1 CIRP^−/−^ mouse died prior to the post-alcohol scan and the second behavior analysis due to technical difficulties encountered during vein cannulation. In addition, behavioral testing failed in one CIRP^−/−^ mouse at the pre-alcohol test, and behavioral testing was not conducted in one WT mouse but the pre-alcohol and post-alcohol scans were completed.

All mice had ad libitum access to food and water throughout the experiment. During the 15 h continuous infusion, mice were awake and were given food pellets and water gels inside the cages to allow easy access to food and water. Those animals that died prior to the second scan and behavioral tests died during vein cannulation. There were no mortality events during continuous infusion or during pre- or post-alcohol scanning periods. A separate cohort of mice (*n* = 3) received 1.5 g per kg alcohol bolus and 1 h later, they were re-anesthetized with 3% isoflurane inhalation, euthanized by CO_2_ asphyxiation and blood collected via cardiac puncture. Blood alcohol concentration (BAC) was measured immediately using a commercially available kit (Pointe Scientific). BAC reached to 37–38 mM (0.17%) at 1 h after the initial bolus dose, which constitutes binge alcohol levels. In an additional cohort of WT and CIRP^−/−^ mice (*n* = 5/group), a test and re-test study was conducted. The same experimental protocol and timeline as described earlier were followed with the exception that all mice were treated with normal saline instead of alcohol.

### Statistical analysis

Data of regional glucose metabolism or measures of behavioral testing were compared separately between conditions and groups. Changes between conditions were assessed by paired *t*-tests and differences between groups were evaluated by unpaired *t*-tests and Mann-Whitney test. Analysis for group and conditions interaction was conducted using two-way analysis of variance (ANOVA). Correlation analysis of regional metabolic values among different regions and with spatial cognition tasks was conducted by Pearson correlation.

## Results

### Relative brain glucose metabolic activity was decreased regionally in mice after binge alcohol

**Fig. 1:**
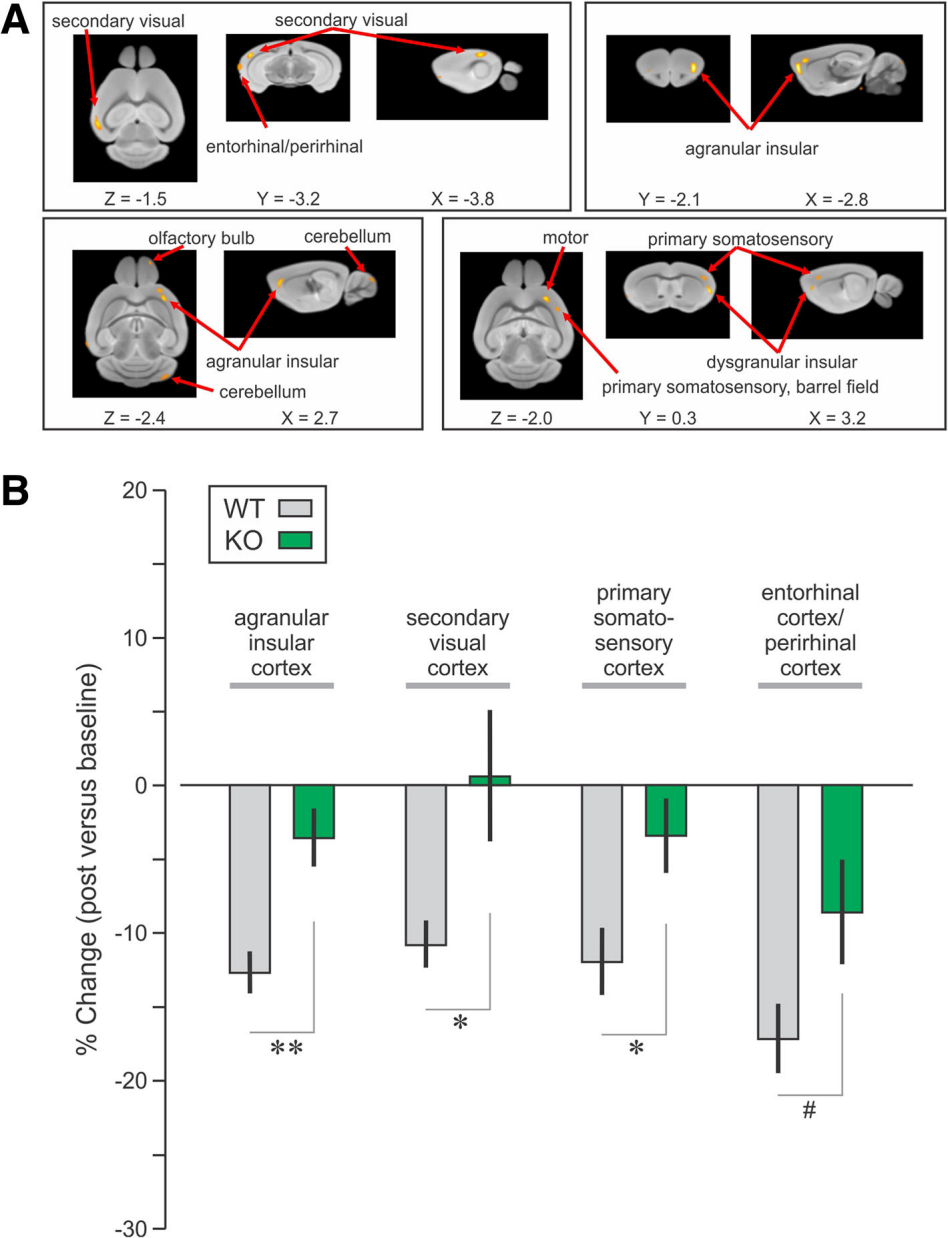
Regional metabolic activity was decreased in the WT mice after binge alcohol and deficiency in CIRP attenuated these decreases in metabolic activities. **a** ^18^FDG-PET images from WT mice were analyzed by SPM8 software. Regions with decreased metabolic activity were aligned onto a standard mouse MRI brain template and regions anatomically identified based on a three-dimensional mouse brain atlas (Paxinos and Franklin 2008). Using *bregma* as the reference point, the Y-coordinate corresponds to the anterior-posterior (AP) distance from *bregma* in the coronal plane, the X-coordinate corresponds to the sagittal or mediolateral (ML) plane, and the Z-coordinate corresponds to the axial or dorsoventral (DV) views of the brain regions. **b** Percent changes in globally normalized metabolic values in WT and CIRP^−/−^ (KO) mice of specific regions identified in the WT group. Data are expressed as mean ± SE; * *P* < 0.05, * *P* < 0.001; # *P* = 0.069, KO vs. WT mice of respective regions

The analysis of WT brains consisted of defining regions of metabolic changes between the pre-alcohol and post-alcohol treatment conditions, by using a paired t-test model with the display parameter (Threshold (T) = 4.297, *P* < 0.001). We found several brain structures that showed decreased glucose metabolism, which were identified based on the 3D mouse brain atlas (Fig. 1a). Specific brain regions, their peak coordinates and *t*-value (actual thresholds) are presented (Tables 2 and 3).

**Table 2:** Regional metabolic activity was decreased in WT mice after binge alcohol. Regions identified by SPM with decreases in metabolic activity in WT mice post-alcohol (*T* = 4.3, *P* = 0.001)

Regions	Anatomical Regions	X (mm)	Y (mm)	Z (mm)	Threshold	Voxels
R11	Agranular insular cortex, dorsal part	2.2	2.1	−2.8	10.07	2677
R13	Secondary visual cortex, lateral area	−3.8	− 3.2	− 1.5	7.04	819
R14	Glomerular layer of the olfactory bulb	1.3	4.8	−2.9	6.93	1567
R15	Entorhinal/perirhinal cortex	−4.7	−3.6	−2.9	6.65	1181
R17	Spinal trigeminal tract	1.8	−7.8	−4.6	6.4	945
R18	Spinocerebellar tract	− 1.5	−8.1	− 5.8	5.86	2714
R19	Primary somatosensory cortex, barrel field	3.2	0.3	−2.0	5.24	142

**Table 3:** Regional metabolic activity was decreased in WT mice after binge alcohol. Regions identified using two way ANOVA by SPM with decreases in metabolic activity in WT mice post-alcohol (*T* = 2.4, *P* < 0.01)

Regions	Anatomical Regions	X (mm)	Y (mm)	Z (mm)	Threshold	Voxels	*P*
R1	Olfactory bulb, glomerular layer	−1.9	3.8	−2.4	3.99	429	0.001
R2	Agranular insular cortex, ventral part	1.7	2.6	−2.6	3.38	936	0.001
R3	Cerebellum, third lobule of cerebellar vermis	−1.5	−5.9	−2.9	3.23	297	0.001
R4	Posteriomedial cortical amygdaloid area	−2.7	−3.3	−5.3	3.22	243	0.001
R5	Hippocampus, fimbria	−2.7	− 2.0	− 3.9	3.22	948	0.001
R6	Primary visual cortex	−4.1	−4.0	−1.5	3.15	359	0.002
R7	Primary somatosensory cortex, barrel field	2.9	0.3	−2.0	3.13	596	0.002

### CIRP deficiency attenuated the decrease in brain metabolic activity observed in WT mice after binge alcohol

We observed, using the region-of-interest analysis, that the percent (%) changes of the globally normalized metabolic values were decreased to a smaller degree in CIRP^−/−^ mice compared to WT mice. Among these regions, the % changes were significantly smaller in CIRP^−/−^ mice for the following cortical regions: agranular insular, secondary visual, primary somatosensory, and reached a trend level in entorhinal/perirhinal cortex (Fig. 1b, Table 4). In a correlation analysis where the data were combined between WT and CIRP^−/−^ mice (*n* = 22), changes in regional metabolic values in the entorhinal/perirhinal cortex correlated with those in agranular insular cortex and primary somatosensory cortex (*r* > 0.5; *P* < 0.02).

**Table 4:** Regional metabolic activity in regions identified in the WT mice after binge alcohol. Percent changes in regional metabolic activity in the WT and CIRP^−/−^ mice treated with alcohol or normal saline (mean ± SE). * *P* < 0.05 and ** *P* < 0.01 between pre- and post-saline treatment within groups

Regions	Anatomical Regions	Alcohol			Saline		
		WT (% change)	KO (% change)	*P*	WT (% change)	KO (% change)	*P*
R11	Agranular insular cortex, dorsal part	−12.7 ± 1.4	−3.5 ± 1.9	0.001	−3.6 ± 3.1	−1.3 ± 3.1	0.84
R13	Secondary visual cortex, lateral area	−10.8 ± 1.6	0.6 ± 4.4	0.029	1.0 ± 5.0	7.7 ± 7.1	0.46
R14	Glomerular layer of the olfactory bulb	−16.1 ± 2.4	−15.5 ± 2.8	0.887	10.6 ± 10.3	−12.5 ± 5.1	0.08
R15	Entorhinal/perirhinal cortex	−17.2 ± 2.4	−8.6 ± 3.5	0.069	−11.1 ± 3.9	−7.2 ± 7.1	0.64
R17	Spinal trigeminal tract	−15.8 ± 2.3	−16.5 ± 3.2	0.501	−2.9 ± 6.7	−19.0 ± 5.1	0.09
R18	Spinocerebellar tract	−31.7 ± 4.9	− 32.9 ± 6.1	0.878	0.03 ± 13.2	−7.2 ± 14.0	0.72
R19	Primary somatosensory cortex, barrel field	−11.9 ± 2.3	−3.4 ± 2.4	0.02	−10.3 ± 1.9**	−10.9 ± 3.7*	0.88

### CIRP deficiency reversed the decrease in brain metabolic activity observed in WT mice after binge alcohol

**Fig. 2:**
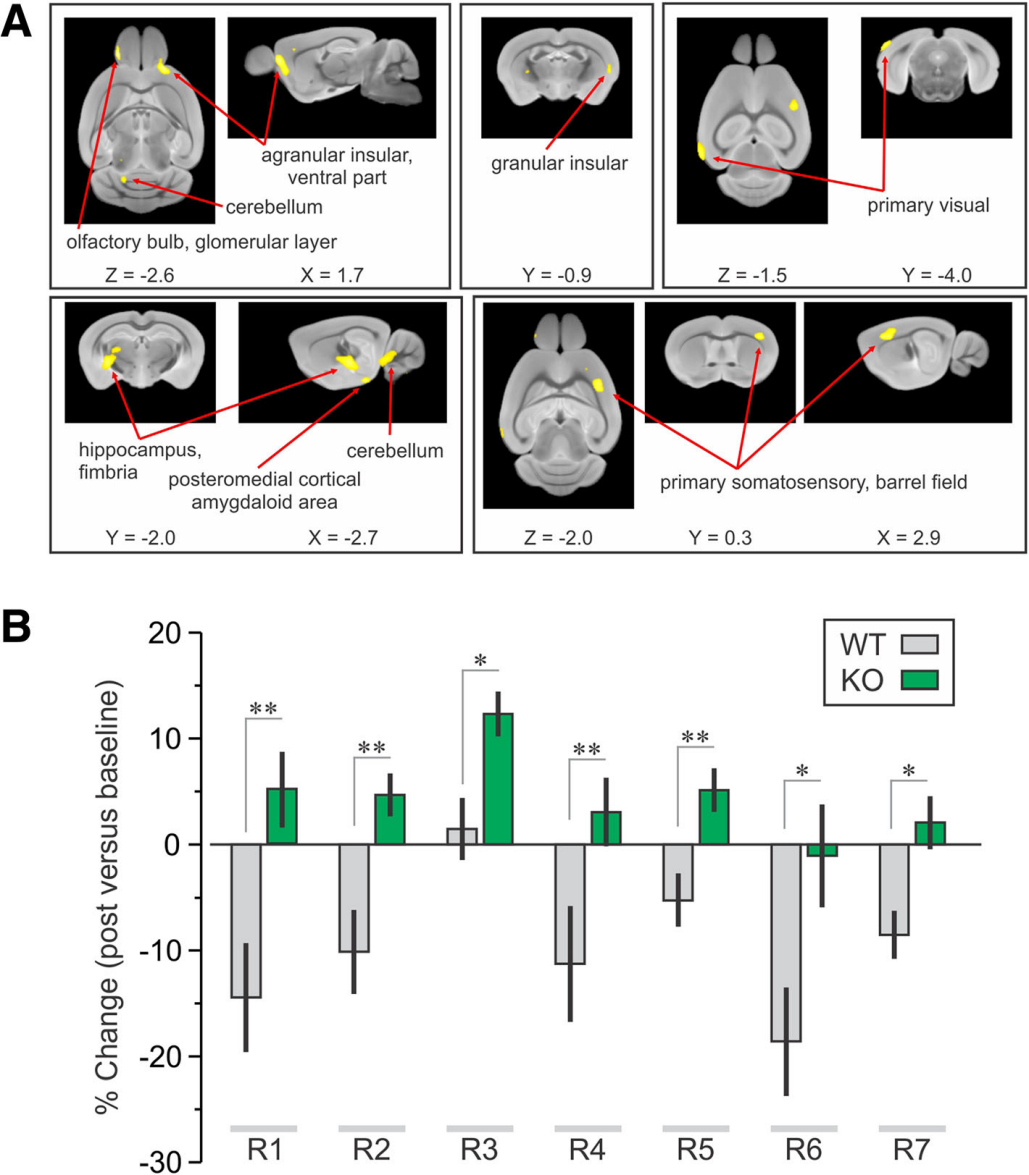
Deficiency in CIRP reversed the decreased regional metabolic activity in the WT mice after binge alcohol. **a** Regions identified by SPM analysis using two way ANOVA in which metabolic activity was decreased in WT mice but increased in KO mice during post-alcohol were aligned to mouse MRI brain template and regions anatomically identified based on a three dimensional mouse brain atlas (Paxinos and Franklin 2008). **b** Percent changes in globally normalized metabolic values decreased in WT mice but increased in CIRP^−/−^ (KO) mice over specific regions revealed by the two-way ANOVA. The regions are as follows: R1-Olfactory bulb, glomerular layer, R2-agranular insular cortex, ventral part, R3-cerebellum, 3rd lobule of cerebellar vermis, R4- posteromedial cortical amygdaloid area, R5-hippocampus, fimbria, R6-primary visual cortex, R7-primary somatosensory cortex, barrel field. See Table 2 for the stereotaxic coordinates and anatomic volumes of these regions. Data are expressed as mean ± SE; * *P* < 0.05, ** *P* < 0.005, KO vs. WT mice of respective regions

The percent changes of the globally normalized metabolic values from WT mice and CIRP^−/−^ mice were analyzed in SPM using a two-way ANOVA to examine the effect of group x condition interaction. When the display parameter was set to Threshold = 2.42, *P* < 0.01, the analysis produced 7 regions where metabolic activity decreased in WT mice but increased in CIRP^−/−^ mice (Fig. 2a, b, Table 5). The same 7 regions were also significant with the display parameter set to *P* < 0.005. The two-way ANOVA revealed similar regions to the original analysis including the insular, visual and primary somatosensory cortices. Interestingly, this analysis identified two additional regions, i.e., the hippocampus and the cortical amygdala (specifically, the posteromedial cortical amygdaloid area, PMCoAA). In a correlation analysis when the data were combined between WT and CIRP^−/−^ mice (*n* = 17), strong correlations between regions were identified in treatment induced changes in relative metabolic values. The fimbria of the hippocampus correlated with PMCoAA (r > 0.5; *P* = 0.02), and primary somatosensory cortex, barrel field (r > 0.4; *P* = 0.05).

**Table 5:** Regional metabolic activity in regions identified by the two way ANOVA analysis. Percent changes in regional metabolic activity in the WT and CIRP^−/−^ mice treated with alcohol or normal saline (mean ± SE). ** *P* < 0.01 between pre- and post-saline treatment within groups

Regions	Anatomical Regions	Alcohol			Saline		
		WT (% change)	KO (% change)	*P*	WT (% change)	KO (% change)	*P*
R1	Olfactory bulb, glomerular layer	−14.5 ± 5.1	5.2 ± 3.1	0.004	13.1 ± 12.4	10.5 ± 12.2	0.89
R2	Agranular insular cortex, ventral part	−10.1 ± 4.0	4.7 ± 2.0	0.002	−5.8 ± 4.8	4.7 ± 3.8	0.13
R3	Cerebellum, third lobule of cerebellar vermis	1.5 ± 2.9	12.3 ± 2.1	0.006	−0.3 ± 4.8	10.3 ± 5.4	0.18
R4	Posteriomedial cortical amygdaloid area	−11.3 ± 5.5	3.1 ± 3.2	0.03	1.2 ± 3.8	2.1 ± 6.4	0.91
R5	Hippocampus, fimbria	−5.3 ± 2.5	5.1 ± 2.1	0.004	2.8 ± 4.0	1.1 ± 3.4	0.75
R6	Primary visual cortex	−18.6 ± 5.1	−1.1 ± 4.8	0.022	4.2 ± 5.5	14.4 ± 8.2	0.1
R7	Primary somatosensory cortex, barrel field	−8.5 ± 2.3	2.1 ± 2.5	0.006	−10.0 ± 0.9*	−6.6 ± 3.1	0.34

### Regional brain metabolic activity was similar in WT and CIRP^−/−^ mice after saline control treatment

Normalized glucose metabolic values were computed *post-hoc* in test-retest acquisitions using the same regions that were defined using paired t-test in the WT group (Table 2) and ANOVA in the WT and CIRP^−/−^ groups (Table 3) respectively. The post-hoc analysis for regions in Table 2 showed that regional ^18^FDG uptake values were stable in the WT or CIRP^−/−^ mice (*P* > 0.05, *P* > 0.08, paired t-tests) in all regions except primary somatosensory cortex (barrel field), where the retest values were lower (*P* < 0.008) than those in the test scans (Table 4). Similar analyses for regions in Table 3 were conducted on regions identified in the ANOVA (Table 5). Interestingly, with the exception of the same region, primary somatosensory cortex (barrel field), (*P* < 0.001), no significant difference in percent changes were observed between pre- and post-saline in any other region in the WT or CIRP^−/−^ mice (*P* > 0.05, *P* > 0.12, paired t-test). Nevertheless, no group differences were detected in any regions in terms of percent changes in regional glucose metabolic values between post- and pre-saline conditions in both analyses (*P* > 0.08, unpaired t-tests).

The possible influence of plasma glucose levels on the observed results was also analyzed. This is important not only to indicate rigorous quality control of the study, but also to support the robustness of regionally specific differences in cerebral glucose metabolism detected in the brain between the treatment conditions and animal groups. Indeed, no treatment-related changes were found for the injected dose, plasma glucose, global SUV values before and after correction with plasma glucose concentration in WT (*P* > 0.17, paired t-test) or CIRP^−/−^ (*P* > 0.07) group or in the combined group (*P* > 0.11). No group differences were detected in these variables except in global SUV values corrected for plasma glucose at post-alcohol treatment (*P* < 0.05, unpaired t-test). In the test re-test study, no treatment-related changes were found for the injected dose, plasma glucose, global SUV values before and after correction with plasma glucose concentration in WT (*P* > 0.06, paired t-test) or CIRP^−/−^ (*P* > 0.35) group or in the combined group (*P* > 0.11). No group differences were detected in these variables in the WT (*P* > 0.09, unpaired t-test) and CIRP^−/−^ (*P* > 0.14) groups. No group differences were seen (*P* > 0.07, unpaired t-test) in these variables in terms of % changes between pre- and post-saline conditions. A detailed statistical analysis is shown in Table 6.

**Table 6:** Absolute SUV values corrected for plasma glucose concentration. Global SUV values corrected for glucose levels at post-alcohol between WT and CIRP^−/−^ mice are significant (*P* = 0.034, unpaired t-test). No group differences were seen in global SUV values in percent changes between pre- and post-saline treatment (*P* > 0.07, unpaired test)

	Weight (g)	Injected dose (mCi)	Blood glucose (mg/dl)	Global SUV	Global SUV x blood glucose
Cohort 1					
WT-baseline	25.2 ± 0.53	0.98 ± 0.21	209 ± 10.0	84.62 ± 5.87	16.84 ± 1.68
WT-post-alcohol	23.6 ± 0.43	0.67 ± 0.10	203 ± 11.9	82.86 ± 8.58	14.88 ± 1.05
WT baseline vs. post-alcohol (P value)	0.029	0.218	0.807	0.780	0.171
KO-baseline	28.6 ± 1.11	1.00 ± 0.20	212 ± 19.0	91.48 ± 7.82	18.90 ± 1.60
KO-post-alcohol	27.8 ± 1.23	0.79 ± 0.11	193 ± 7.80	108.47 ± 9.51	21.18 ± 2.42
KO baseline vs. KO post-alcohol (*P* value)	0.181	0.348	0.421	0.072	0.528
WT + KO (baseline)	27.0 ± 0.7	0.99 ± 0.14	211 ± 12.0	88.37 ± 4.98	18.08 ± 1.27
WT + KO (post-alcohol)	25.8 ± 0.81	0.73 ± 0.07	198 ± 6.67	96.28 ± 6.90	18.20 ± 1.52
WT + KO baseline vs WT + KO post-alcohol (P value)	0.009	0.113	0.403	0.146	0.991
Cohort 2					
WT-baseline	23.60 ± 0.81	0.67 ± 0.04	184 ± 11.8	99.32 ± 10.86	16.41 ± 2.31
WT-post-saline	24.40 ± 0.60	0.83 ± 0.04	175 ± 14.6	67.07 ± 7.09	12.09 ± 0.55
WT baseline vs. post-saline (P value)	0.242	0.09	0.73	0.058	0.211
KO-baseline	23.0 ± 0.55	0.61 ± 0.04	190 ± 12.4	71.80 ± 9.29	12.66 ± 0.56
KO-post-saline	23.4 ± 0.40	0.66 ± 0.11	197 ± 9.5	72.57 ± 4.67	13.58 ± 0.72
KO baseline vs. KO post-saline (P value)	0.374	0.678	0.499	0.934	0.346
WT + KO (baseline)	23.30 ± 0.47	0.64 ± 0.03	187 ± 7.8	85.56 ± 8.15	14.53 ± 1.35
WT + KO (post-saline)	23.90 ± 7.56	0.74 ± 0.23	186 ± 9.0	69.82 ± 4.11	12.83 ± 4.54
WT + KO baseline vs WT + KO post-saline (P value)	0.111	0.135	0.377	0.114	0.322

### CIRP deficiency prevented the impaired spatial cognition observed in WT mice after binge alcohol exposure

**Fig. 3:**
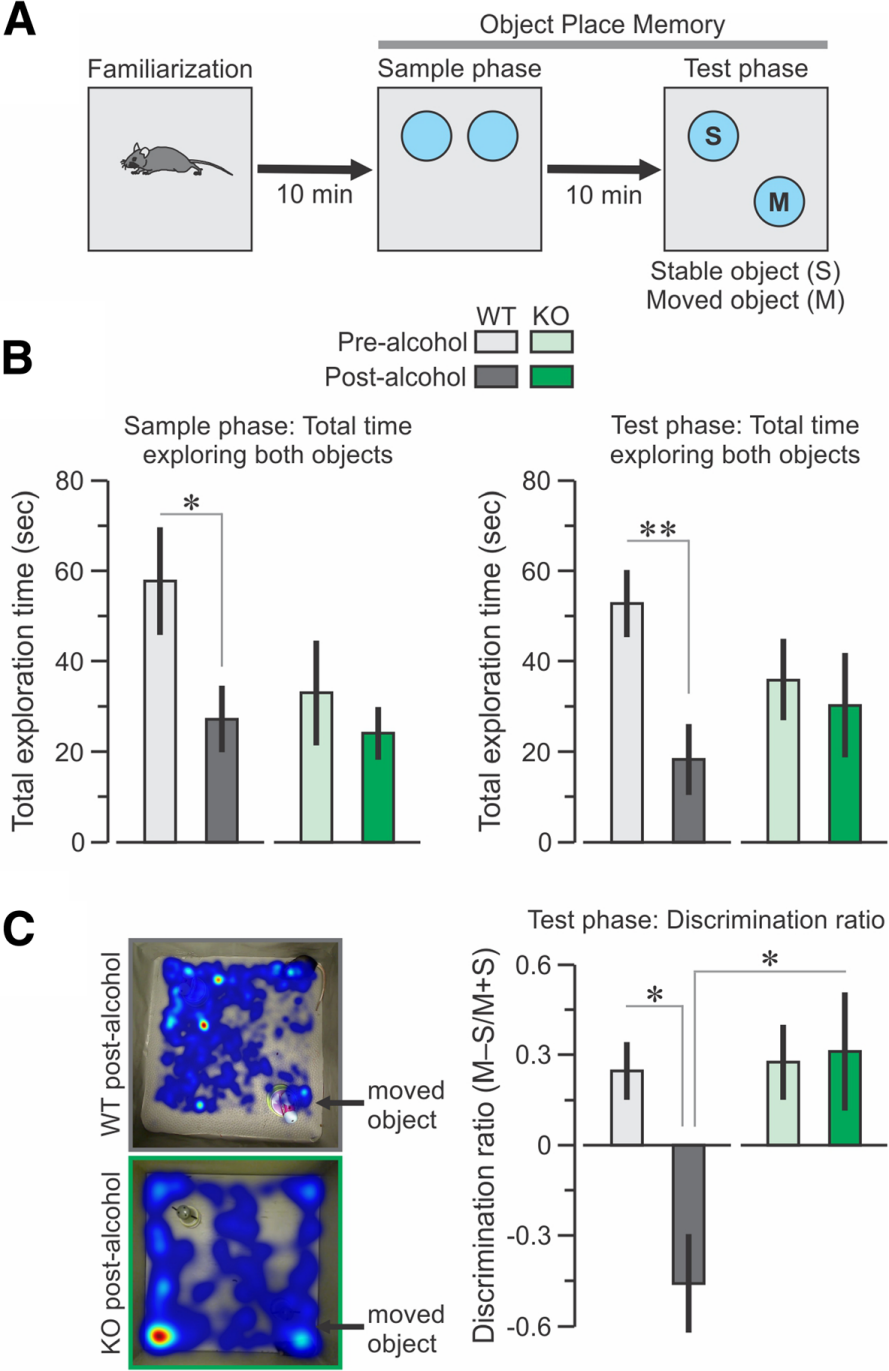
Deficiency in CIRP prevented the decreased spatial cognition observed in the WT mice after binge alcohol. **a** Scheme for the object-place memory (OPM) task in which a mouse explores two objects, with one object being moved in the last session. **b** Total time exploring both objects during sample phase and test phases. **c** *Left*, representative heat maps of WT and CIRP^−/−^ mice exploring the moved object, *Right*, discrimination ratio (moved minus stable object over moved plus stable object [(M-S)/(M + S)]) between WT and CIRP^−/−^ mice at pre-alcohol and post-alcohol conditions. Data are expressed as mean ± SE; * *P* < 0.05; ** *P* < 0.005 by t-test

For the OPM task (Fig. 3), a positive discrimination ratio indicates the preferential exploration of a moved object and spatial memory. The total time exploring objects during the sample phase and test phases were significantly decreased in the WT mice post-alcohol but in the CIRP^−/−^ mice, these times were not altered from the pre-alcohol tests (Fig. 3b). Both WT and CIRP^−/−^ mice had similarly positive discrimination ratios at pre-alcohol. The alcohol treatment did not perturb spatial cognition in CIRP^−/−^ mice as they continued to examine a recently moved object during the OPM task (*T* = 0.15, *P* = 0.87, t test). Conversely, WT mice showed significantly impaired OPM performance (*T* = 3.9, *P* = 8.75 × 10^− 4^, t test) (Fig. 3c). A two-way ANOVA of the discrimination ratios revealed a significant difference by genotype (*F* = 5.22, *P* = 0.027) as well as genotype x alcohol treatment (*F* = 4.21, *P* = 0.021).

### CIRP deficiency inhibited the altered OF behavior shown by WT mice after binge alcohol exposure

**Fig. 4:**
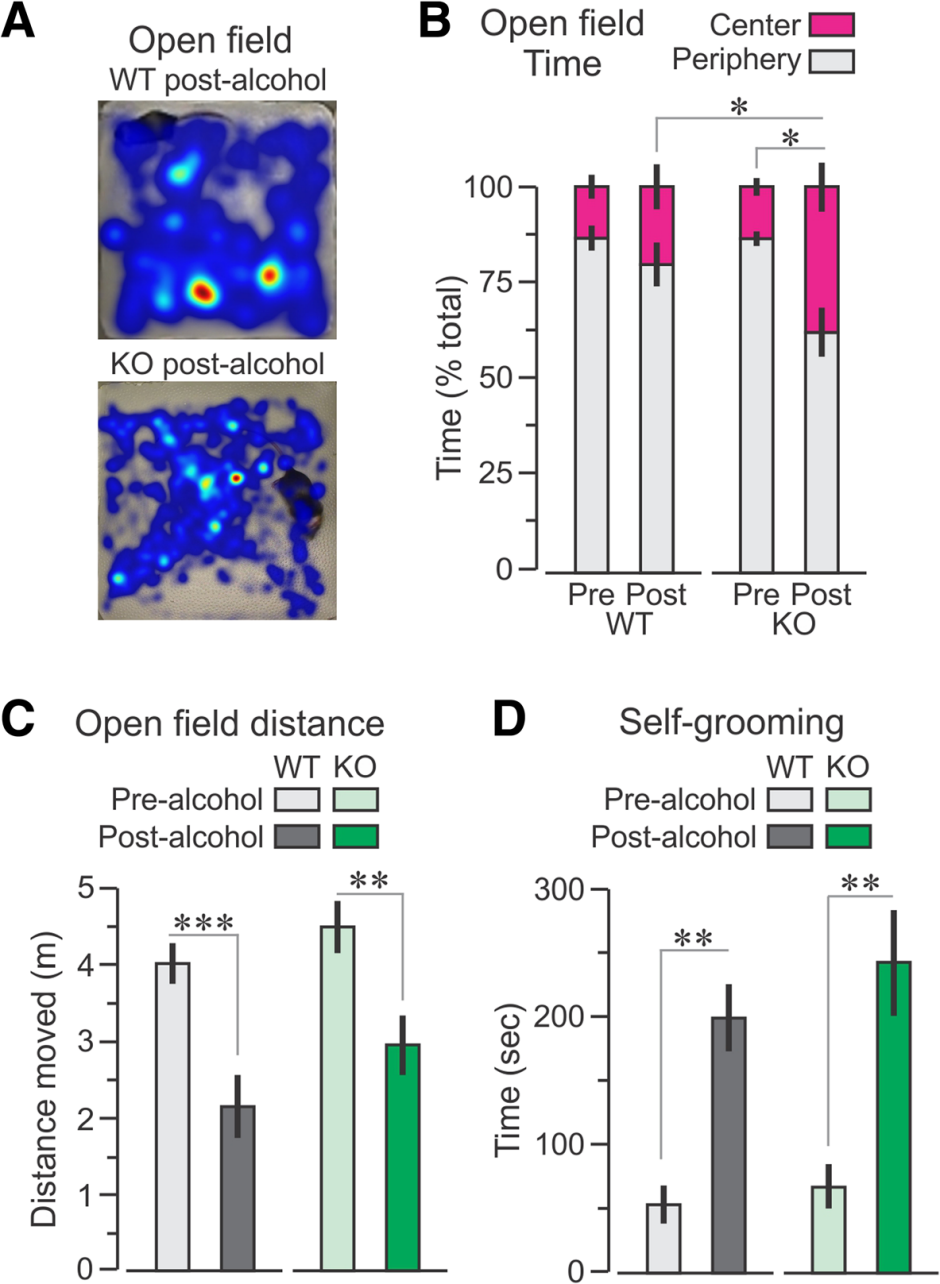
Deficiency in CIRP inhibited the altered open field behavior exhibited by WT mice. **a** Representative heat map of WT and CIRP^−/−^ mice exploring the chamber. **b** Percent time spent exploring either the center or periphery of the chamber. **c** Total distance traversed during the test. **d** Time spent for self-grooming during the OF tasks. Data are expressed as mean ± SE; * *P* < 0.05; ** *P* < 0.005, *** P < 0.001 by t-test

WT and CIRP^−/−^ mice spent similar time in the center and periphery of the chamber at pre-alcohol. However, the alcohol treatment resulted in CIRP^−/−^ mice displaying higher exploration of the center of the OF than the WT group, demonstrating their familiarity to the chamber (Fig. 4b). The total distances traversed were significantly decreased from pre-alcohol to post-alcohol in both WT and CIRP^−/−^ mice, but the decrease was smaller in CIRP^−/−^ mice (Fig. 4c). A two-way ANOVA of the distance demonstrated no difference by genotype (*F* = 3.41, *P* = 0.07), but significant variance by alcohol treatment (*F* = 24.36, *P* = 1 × 10^− 5^) as well as genotype x treatment (*F* = 13.56, *P* = 2.2 × 10^− 5^). Additionally, both groups showed increased levels of self-grooming after the alcohol treatment (Fig. 4d).

### Regional metabolic activity is significantly correlated with spatial cognition after binge alcohol exposure

**Fig. 5:**
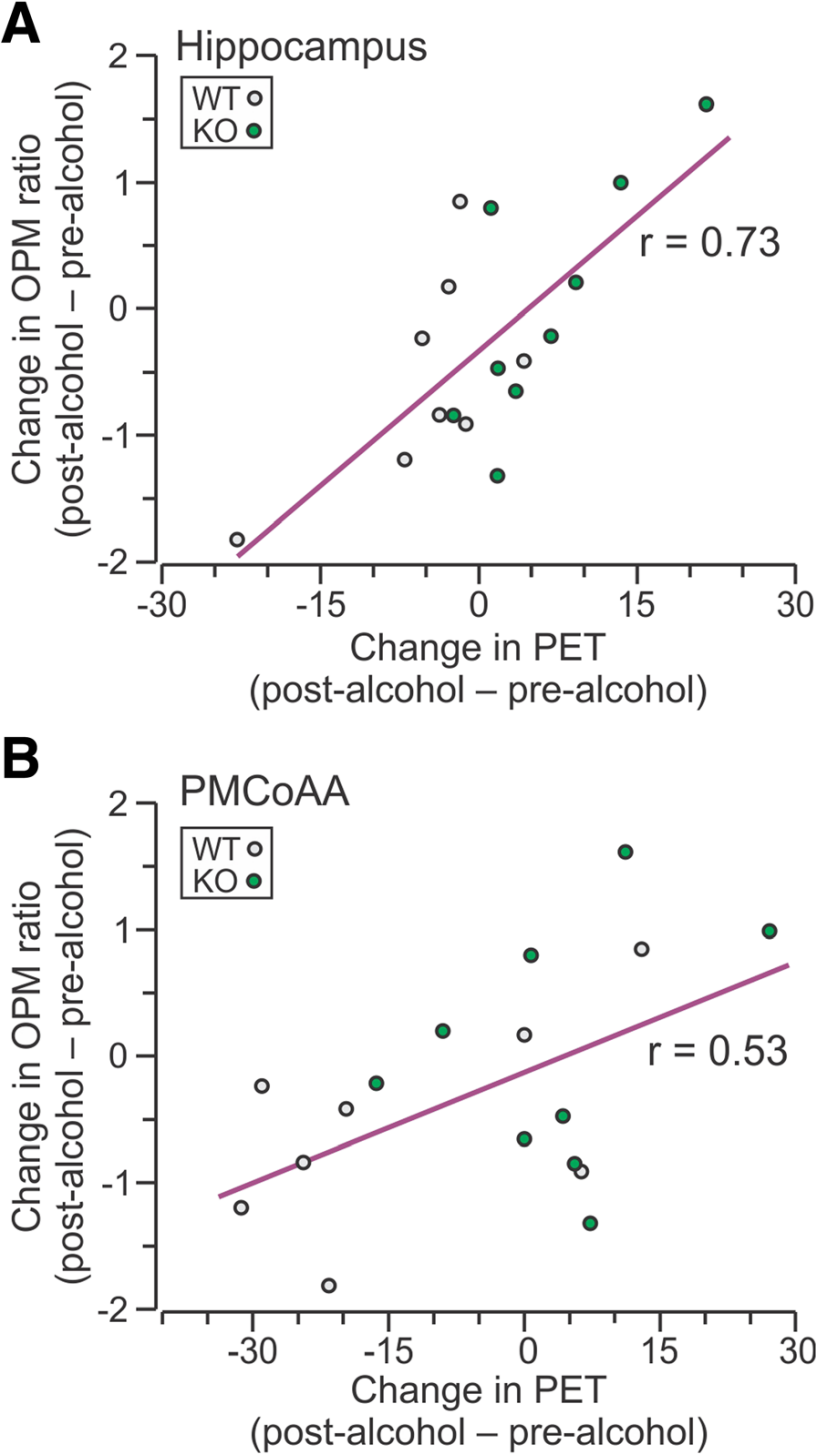
Regional metabolic activity is correlated with spatial cognition after binge alcohol. Pearson correlation analysis of percent changes in globally normalized metabolic values in the WT (open circles) and CIRP^−/−^ mice (closed circles) with changes in OPM ratio of the hippocampus (*r* = 0.73, *P* < 0.001) and posteromedial cortical amygdaloid area (PMCoAA; *r* = 0.53, *P* = 0.03)

In a correlation analysis where the data were combined between WT and CIRP^−/−^ mice (*n* = 17), there was a strong correlation between the changes in discrimination ratio and the percent changes in metabolic activity the fimbria of the hippocampus (*r* = 0.73, *P* < 0.001) and in the PMCoAA (*r* = 0.53, *P* = 0.03) (Fig. 5).

## Discussion

Binge level of alcohol administration in healthy non-alcoholic subjects showed decreased brain glucose metabolism using ^18^FDG-PET (Volkow et al. 2006) but similar studies have not been done in rodent models of binge alcohol exposure. The objective of the current study was to first identify regions in WT mice where brain glucose metabolism decreased due to binge alcohol exposure, and then determine whether those specific regions were less suppressed in the CIRP^−/−^ mice after alcohol. In order to address the first objective, SPM analysis with paired *t*-test using general linear models were performed on the data obtained from the WT mice during pre- and post-alcohol. This model produced a set of brain regions of ^18^FDG uptake in the WT mice showing highly significant decreases in globally normalized metabolic activity. The regions were overlaid onto a mouse MRI brain template, and identified using an anatomical mouse brain atlas (Paxinos and Franklin 2008). To address the second objective, globally normalized metabolic values of WT and CIRP^−/−^ brain regions were analyzed post hoc in each of the regions identified by SPM analysis. The initial analysis revealed marked differences between the two animal groups in several regions, including the temporal (secondary visual) cortex and a trend difference in the limbic (entorhinal/perirhinal) cortex. This simple strategy tested the main hypothesis of neuroprotection in CIRP^−/−^ mice versus WT mice following binge alcohol exposure. The SPM analysis was then performed using two-way ANOVA to reveal the effect of group X condition interaction. The two-way ANOVA revealed similar regions of ^18^FDG uptake to the original analysis including the insular, visual and primary somatosensory cortices. Interestingly, this analysis identified additional regions such as the hippocampus and the cortical amygdala.

The present study showed that binge alcohol exposure in WT mice leads to decreases in relative brain glucose (^18^FDG) uptake within several neocortical areas, including the temporal lobe. The medial temporal lobe consists of the hippocampal region and the adjacent perirhinal, entorhinal and the parahippocampal cortices, which are essential for spatial and episodic memory (Squire et al. 2004). In rodent models, dysfunction of these regions results in impaired object place memory (Faust et al. 2013). Acute ethanol exposure can also result in deficient spatial reference memory (Matthews et al. 1995; Matthews et al. 1999; Matthews and Morrow 2000). We found that binge alcohol-exposed WT mice showed abnormalities when exploring a moved object in the OPM task and when exploring an open field. In contrast, similar exposure to alcohol in CIRP^−/−^ mice did not lead to impairments in the OPM and the OF tasks. Pearson correlation analysis between the changes in relative metabolism in the 7 regions identified by two-way ANOVA and the changes in OPM ratio showed strong correlations in the hippocampus and the cortical amygdala. The revelation that the metabolic activity is decreased in the hippocampus after binge alcohol further strengthens the observation of the significant decrease in the OPM ratio in WT mice. The data also revealed a strong correlation between regions identified in the two-way ANOVA analysis. Interestingly, the changes in regional metabolic activities in the hippocampus correlated with those in the cortical amygdala. Therefore, the current study strongly suggests that binge alcohol exposure decreases metabolic activity in brain regions associated with memory and cognition of WT mice, whereas these activities are relatively preserved in the CIRP^−/−^ mice, indicating a crucial role of CIRP during binge alcohol exposure.

Since its discovery in 1997, diverse functions of CIRP have been increasingly recognized, from being an RNA chaperone, to a mediator of inflammation (Nishiyama et al. 1997; Sheikh et al. 1997; Xue et al. 1999; Gualerzi et al. 2003; Wellmann et al. 2004; Morf et al. 2012; Qiang et al. 2013). CIRP is expressed at low levels in nearly all tissue, and upon cellular stress, it can be translocated to the cytosol where it binds to 3′ regions of target mRNAs to either increase or decrease their translation. This interaction regulates cell proliferation, survival, apoptosis and the circadian rhythm. While CIRP may function as a protective protein intracellularly, it can be released from cells and the extracellular CIRP may work as a DAMP that promotes inflammation (Qiang et al. 2013; Rajayer et al. 2013; Zhou et al. 2013). Since in the present study, CIRP^−/−^ mice were protected from the decreases in metabolic brain activity and cognitive deficits that occur after binge alcohol exposure, it can be speculated that the observed protection is due to the elimination of either the intracellular or extracellular CIRP.

A major limitation to our study is that we did not discriminate whether the observed decrease in metabolic activity in WT mice was due to extracellular or intracellular effects of CIRP. A previous study that included surgical intensive-care patients, as well as rodent models of hemorrhagic shock and sepsis, found detectable levels of CIRP protein in the blood (Qiang et al. 2013). Blocking CIRP, either by neutralizing antibody or the use of CIRP^−/−^ mice, showed decreased inflammation and improved survival in several rodent models of inflammatory diseases including hemorrhagic shock and sepsis (Qiang et al. 2013; Godwin et al. 2015; Cen et al. 2016). In vitro experiments with macrophage cells exposed to hypoxia/reoxygenation, or LPS stimulation, have shown that CIRP is translocated from nucleus to cytosol and secreted into the medium (Qiang et al. 2013). In addition, exogenous administration of recombinant murine CIRP in healthy rats increased markers of liver injury and inflammatory cytokines (TNF-α, IL-6 and HMGB1), indicating that extracellular CIRP is an inflammatory mediator that can trigger inflammation under stress conditions (Qiang et al. 2013). The extracellular action of CIRP is mediated by TLR4/MD2 and acts as a DAMP to promote inflammation (Qiang et al. 2013). Released CIRP has been shown to induce endothelial cell pyroptosis and initiate adaptive T-cell responses (Yang et al. 2016; Bolognese et al. 2018). CIRP can also be released from BV2 microglia cells in response to alcohol (Rajayer et al. 2013). These previous findings strongly suggest that extracellular CIRP could be responsible for the binge alcohol-induced regionally specific brain hypoactivity leading to impaired cognition. A behavioral limitation is that the testing was solely selected based on previous reports (Garcia-Moreno and Cimadevilla 2012). Specific behavioral analysis related to the regions identified in the ^18^FDG-PET analysis should provide insights into the role of CIRP and behavioral dysfunction in binge alcohol exposure.

It is important to examine several experimental factors that may impact the methodology of PET imaging acquisition and analysis. With the exception of a slight decrease in body weight in the post-alcohol WT mice (*P* = 0.03), there were no treatment related changes in whole blood glucose values, injected dose or global SUV values before and after correction with plasma glucose concentrations in either the WT mice, CIRP^−/−^, or in the combined group. No group differences were detected in these variables except in global SUV values corrected for plasma glucose levels at post-treatment (P = 0.03). These observations confirmed adequate quality control for the imaging portion of the study, and justified the use of globally normalized metabolic values in the analysis of the imaging data. Of note, it is not useful to show specific metabolic characteristics in individual animals due to the limited signal to noise ratios present in each ^18^FDG-PET image. Therefore, regionally specific effects can be detected only by performing brain-mapping analysis with SPM as shown in this study. We focused on the group x treatment interaction to interrogate the main hypothesis of the study that brain glucose metabolism is less suppressed in CIRP^−/−^ mice than WT mice following binge alcohol exposure.

It can be speculated that the difference between pre- and post-alcohol scans are due to simple repetition effects or multiple anesthesia exposures, and not specific for ethanol. To this end, a test-retest study was performed in WT and CIRP^−/−^ mice that followed the same experimental protocols, but were treated with saline in both groups. No treatment-related changes were observed in WT or CIRP^−/−^ group in regard to the injected dose, plasma glucose or global SUV values before and after plasma glucose correction. No group differences were detected in these variables in the WT and CIRP^−/−^ groups, nor were there differences in terms of % changes between pre- and post-saline conditions. The regional metabolic values were stable in all regions except the primary somatosensory cortex (barrel field) in pre- and post-saline in both groups. Nevertheless, no differences were observed between groups for percent changes in the test/retest saline group in any of the regions identified in the alcohol treated groups. Therefore, it is highly unlikely that the differences observed between pre- and post-alcohol scans are simply due to repetition effects.

For the initial behavior analysis, the light-dark cycles were reversed so that the testing was done during the dark circadian period. It can be speculated that the reverse cycle impacted the outcome of the study. However, in order to conduct behavioral studies, it was important to reverse their light-dark cycles, otherwise, the experiment had to be done in the night, i.e., during their day. Since our objective was to correlate the microPET data with the behavior, the behavior tasks were conducted in conjunction with the microPET scans, i.e., 9:00 AM. After the basal behavior and basal scan, mice were kept in the microPET suite for 24–30 h undisturbed with the normal 12-h light/dark cycles. Afterwards, the mice underwent alcohol infusion in the aid of a mouse harness so that they were again undisturbed for 15 h with the normal 12-h light/dark cycles. It is expected that stress of the reverse light-dark cycle and sleep deprivation could similarly impact both groups. Therefore, it is highly unlikely that the changes seen in the behavior between groups and conditions are simply due to experimental stress. In addition, the initial behavioral testing was done prior to IP injection of FDG to minimize radioactivity exposure to the personnel. After 15 h, mice underwent behavior during FDG uptake to prevent time lapse between behavior and microPET scans post-alcohol. Since the same experimental procedure was followed for both groups, the IP injection could not have impacted the observed changes between groups and conditions. Separate experiments conducted each for behavior and microPET scan with the two groups could have circumvented these experimental technical discrepancies encountered in this study. However, choosing to conduct both behavior and FDG-PET in the same mice allowed to perform the correlational analysis between the two parameters which was the fundamental reason for the current experimental design.

The dose of 6 g/kg alcohol could be considered as too high dose to model binge alcohol drinking. However, mice were injected with 1.5 g/kg alcohol bolus for a period of 20 min. This initial dose is the maximum tolerated bolus dose for a mouse and at 1 h after the alcohol bolus, the blood alcohol was 38 mM which constituted the binge alcohol level. Although continuous infusion for 15 h at 300 mg/kg/h totals the alcohol dose to 6 g/kg, the actual blood alcohol level is expected to decrease even from the bolus dose due to rapid metabolism of alcohol in mice. In the future, we plan to use additional lower doses of bolus to ascertain the minimum dose necessary to influence brain hypoactivity and impaired cognition.

The primary objective of this study was to test the hypothesis that CIRP^−/−^ mice have a neuroprotective effect when compared to WT mice in the context of binge alcohol exposure. In the present study we have fulfilled our objective with two approaches: (1) we have identified hypometabolic brain regions in WT mice whose neurobiological substrates remain intact; (2) we have identified hypometabolic brain regions using ANOVA to tease out the interaction between WT and CIRP^−/−^ mice. Regional metabolic values extracted from these sets of regions were then compared across the experimental conditions and animal groups. Both approaches revealed regional brain metabolic decreases in the WT that were less suppressed metabolically in the CIRP^−/−^ mice after binge alcohol exposure. Further studies are needed to replicate our findings in large samples, and to investigate possible mechanisms of spatial co-variation using more powerful multivariate brain mapping analysis.

## Conclusion

These data suggest that extracellular CIRP released from brain cells could be responsible for brain metabolic hypoactivity and impaired cognition in binge alcohol exposure. Blocking released CIRP from brain cells could be a potential therapeutic strategy for binge alcohol drinking.

## Abbreviations

^18^FDG: 18-Fluorodeoxyglucose
BAC: Blood alcohol concentration
CIRP: Cold inducible RNA binding protein
CIRP^-/-^: Mice deficient in CIRP
DAMP: Danger associated molecular pattern
FOV: Field of view
HMGB1: High mobility group box 1
IL-6: Interleukin-6
mCi: milliCurie
NIAAA: National Institute of Alcohol Abuse and Alcoholism
OF: Open field
OPM: Object place memory
PET: Positron emission tomography
PMCoAA: Posteriomedial cortical amygdaloid area
SPM: Statistical parametric mapping
SUV: Standard uptake value
TNF-α: Tumor necrosis factor-alpha
WT: Wild type
